# Early life child protection contacts and developmental risk at age five: a whole-of-population cohort study of 479,413 children in two Australian states.

**DOI:** 10.23889/ijpds.v9i5.2639

**Published:** 2024-09-18

**Authors:** Nicole Satherley, Andrew Sporle

**Affiliations:** 1 iNZight Analytics; 2 University of Auckland

## Introduction

Children who experience maltreatment have worse health and development outcomes than other children. Early prevention relies on opportunities to respond to system contacts that reliably indicate the population burden of children’s future developmental risk.

## Objectives and Approach

We quantified the population burden of developmental vulnerability at age five by the type, timing, and frequency of child protection contacts before school, among children in two Australian states. We used linked whole-population births, child protection and Australian Early Development Census (AEDC) data (2009-2018 cycles) in New South Wales (NSW) and South Australia (SA).

## Results

56,650/398,702 (14%) NSW and 12,617/80,731 (16%) SA children had ≥1 child protection contacts before school. The risk of developmental vulnerability on ≥1 domains was lowest in the no child protection group (NSW, 17-18%; SA, 19%), with higher risks in the child protection report (NSW, 28-29%; SA, 32-35%) through to the OOHC (NSW, 35-38%; SA, 39-50%) groups, with a similar pattern for the risk of medically diagnosed conditions. Children with only one child protection report before school had a higher developmental risk than the no child protection group (NSW, 34% versus 21%; SA, 42% versus 24%).

## Conclusions/Implications

Even a single child protection report in the first 2000 days of children’s lives was a robust indicator of developmental risk at age five, with higher developmental risks among children with more serious child protection contacts before school. Child protection reports represent an under-utilised asset to inform early universal and targeted support from health, human and early education services.

